# Hybrid dynamic/static method for large-scale simulation of metabolism

**DOI:** 10.1186/1742-4682-2-42

**Published:** 2005-10-04

**Authors:** Katsuyuki Yugi, Yoichi Nakayama, Ayako Kinoshita, Masaru Tomita

**Affiliations:** 1 Institute for Advanced Biosciences, Keio University, Fujisawa, Kanagawa, 252–8520, Japan.

## Abstract

**Background:**

Many computer studies have employed either dynamic simulation or metabolic flux analysis (MFA) to predict the behaviour of biochemical pathways. Dynamic simulation determines the time evolution of pathway properties in response to environmental changes, whereas MFA provides only a snapshot of pathway properties within a particular set of environmental conditions. However, owing to the large amount of kinetic data required for dynamic simulation, MFA, which requires less information, has been used to manipulate large-scale pathways to determine metabolic outcomes.

**Results:**

Here we describe a simulation method based on cooperation between kinetics-based dynamic models and MFA-based static models. This hybrid method enables quasi-dynamic simulations of large-scale metabolic pathways, while drastically reducing the number of kinetics assays needed for dynamic simulations. The dynamic behaviour of metabolic pathways predicted by our method is almost identical to that determined by dynamic kinetic simulation.

**Conclusion:**

The discrepancies between the dynamic and the hybrid models were sufficiently small to prove that an MFA-based static module is capable of performing dynamic simulations as accurately as kinetic models. Our hybrid method reduces the number of biochemical experiments required for dynamic models of large-scale metabolic pathways by replacing suitable enzyme reactions with a static module.

## Background

Recent progress in high-throughput biotechnology [1-3] has made advances in understanding of cell-wide molecular networks possible at the systems level [4,5]. To reconstruct cellular systems using the high-throughput data that are becoming available on their components, computer simulations are being revisited as an integrative approach to systems biology. Mathematical modelling of biochemical networks has been attempted since the 1960s, and before genome-scale pathway information became available, they mostly employed numerical integration of ordinary differential equations for reaction rates [6-10]. This kind of dynamic simulation model provides the time evolution of pathway properties such as metabolite concentration and reaction rate. To create accurate simulations, dynamic models require kinetic parameters and detailed rate-laws such as the MWC model [11] and those derived using the King-Altman method [12]. However, with few exceptions such as human erythrocyte metabolism [13,14], it is virtually impossible to collect a complete set of kinetic properties for large-scale metabolic pathways. Therefore, the applicability of the dynamic method has been limited to relatively small pathways.

Another approach, such as metabolic flux analysis (MFA) using stoichiometric matrices, has been employed for large-scale analyses of metabolism [4,15,16]. Assuming a steady-state condition, MFA provides a flux distribution as the solution of the mass balance equation without the need for rate equations and kinetic parameters [16,17]. Since it is a "static" approach, the ability of MFA to predict the dynamic behaviour of metabolic pathways is limited. It provides a snapshot of a certain pathway in a single state, but is insufficient to predict the dynamic behaviour of metabolism [18]. Recently, this approach was extended to allow the prediction of dynamic behaviour. This extension, dynamic flux balance analysis (DFBA) [19], provides optimal time evolution based on pre-defined constraints, including kinetic rate equations. However, this extension was not intended to reduce the masses of information necessary for developing dynamic cell-scale simulation models. In addition, this DFBA study did not define the criteria for segmenting a whole metabolic pathway into parts defined by kinetic rate equations and a stoichiometric model. Therefore this effort does not suffice as a generic modelling approach.

Here we propose a method for dynamic kinetic simulation of cell-wide metabolic pathways by applying the kinetics-based dynamic method to parts of a metabolic pathway and the MFA-based static method to the rest. Because the static module does not require any kinetic properties except the stoichiometric coefficients, this method can drastically reduce the number of enzyme kinetics assays needed to obtain the dynamic properties of the pathway. We have evaluated the accuracy of the hybrid method in comparison to a classical dynamic kinetic simulation using small virtual pathways and an erythrocyte metabolism model.

## Results

### Evaluation of errors

**Figure 1:**
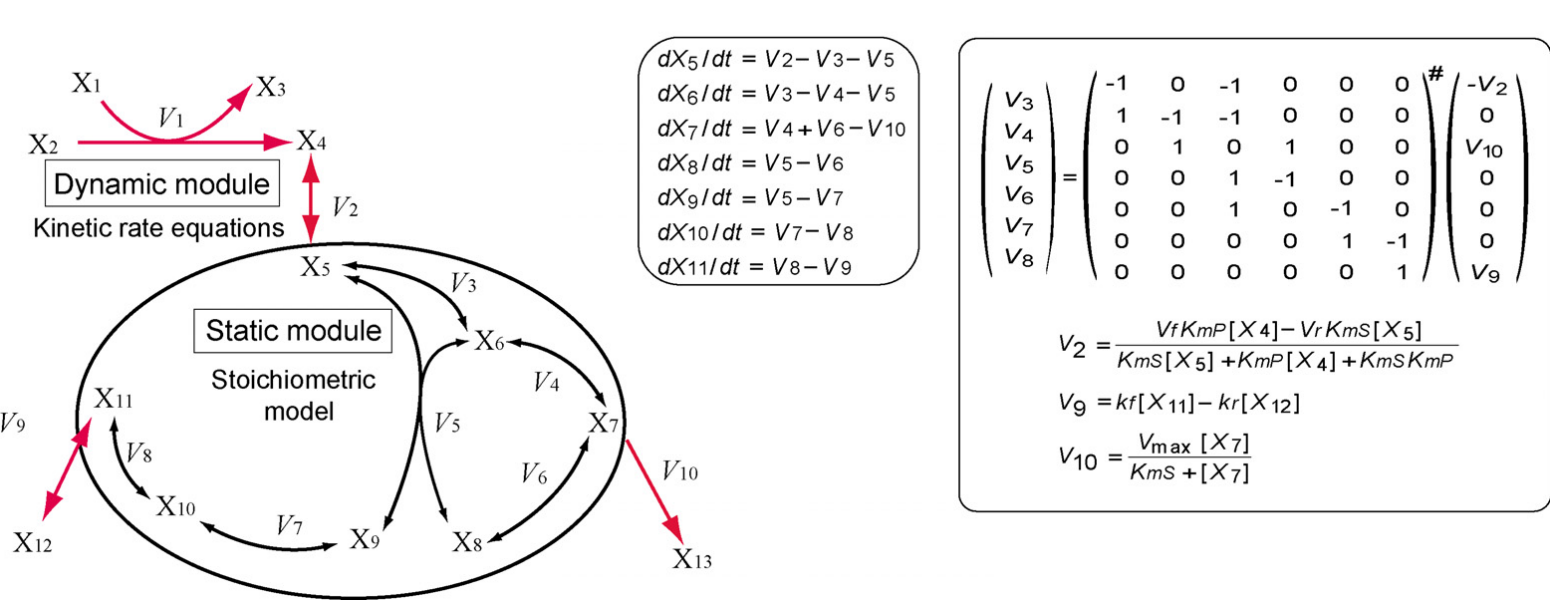
**Summary of the hybrid method**. (i) In the dynamic module (V_1_, V_2_, V_9_, and V_10_), the rate equations provide the reaction rates. (ii) In the static module, the reaction rate distribution (V_3_, V_4_, V_5_, V_6_, V_7_, and V_8_) is calculated from the matrix equation at the right, which corresponds to **v = S^#^b. S^# ^**denotes the Moore-Penrose pseudo-inverse of **S**. (iii) Numerical integration of all the reaction rates (V_1_-V_10_) determines the concentrations of the metabolites (X_1_-X_13_). The metabolites X_5_, X_7_, and X_11 _are at the boundary.

**Figure 2:**
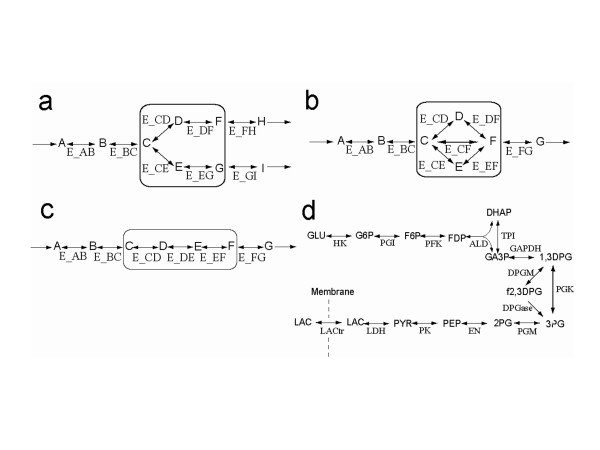
**Hypothetical pathways for simulation experiments**. Simple pathway models employed to evaluate the accuracy of the hybrid method in comparison with conventional kinetic simulation. The reactions in the boxes were replaced with a static module in the hybrid models. (a) A pathway model with an over-determined static module. (b) A model including an underdetermined static module. (c) A simple linear pathway model. (d) A pathway map of the glycolysis model [13, 20]. See Tables 4 and 5 in Additional file 1 for the abbreviations of the metabolites and the enzymes, respectively.

**Figure 3:**
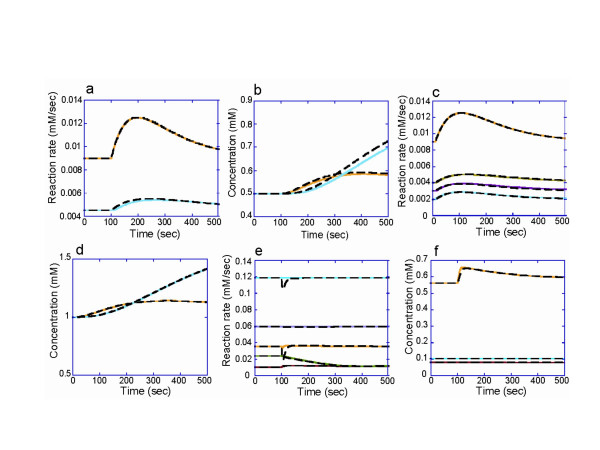
**Comparisons of time courses produced by dynamic and hybrid models**. The coloured lines and the broken black lines represent the time courses calculated by dynamic and hybrid models, respectively. Refer to Fig. 2 for pathway nomenclature. The hybrid model in Fig. 2a yielded similar time courses of change in the reaction rates and the metabolite concentrations to the corresponding dynamic model. (a) The reaction rates of E_BC (yellow) and E_DF (blue). (b) The concentrations of compounds D (yellow) and H (blue). The time courses of the pathway model in Fig. 2b were also in agreement with the dynamic model. (c) The reaction rates of E_BC (yellow), E_CF (green), E_CE (red), and E_CD (blue). (d) The concentrations of compounds E (yellow) and H (blue). The results of these models were also in good agreement for the erythrocyte model. (e) The reaction rates of the hybrid model differed only slightly from those of the dynamic model. The lines in blue, purple, yellow, green, and red denote the reaction rates of GSSGR, G6PDH, TK2, TA and R5PI, respectively. (f) The hybrid and dynamic models yielded almost identical time courses in the concentrations of metabolites such as X5P (yellow), GSSG (blue), and NADP (red).

The hybrid simulation method integrates the two types of simulation method within one model: the static module comprises enzymatic reactions without their kinetic properties and the dynamic module covers the rest of the pathway, thereby enabling the static module to be calculated in a quasi-dynamic fashion (Figure 1). At steady-state, a hybrid model of a hypothetical pathway that included an over-determined static module (Figure 2a) yielded an almost identical solution to a dynamic model of the pathway. The reaction rates were calculated by numerical integration of the rate equations. We employed the errors between the dynamic and hybrid models in the first integration step as an index to estimate the accumulation of errors in the subsequent integration steps (one-step error; see Methods for a detailed definition). The one-step error was 8.592 × 10^-16 ^of the maximum for the reaction rates. All the metabolite concentrations in the hybrid model were identical to those in the dynamic model (Table 1). When the concentration of metabolite A was increased two-fold, the hybrid and the dynamic models displayed similar time evolutions (Figure 3a and 3b). The maximum one-step errors after this perturbation were 4.000 × 10^-11 ^and 8.889 × 10^-6 ^for metabolite concentrations and reaction rates, respectively (Table 1).

**Table 1:** Errors between the dynamic model and the hybrid model of the pathway shown in Fig. 2a. The maximum errors were measured within one numerical integration step. "Perturbation" denotes whether the errors were measured under a steady-state condition (-) or after a two-fold increase of metabolite A (+)

	Perturbation	Maximum error (concentration)	Maximum error (reaction rate)
Boundary	-	0	0
	+	8.000 × 10^-11 ^(C)	0
Static part	-	0	8.592 × 10^-16 ^(E_CD)
	+	4.000 × 10^-11 ^(D,E,F,G)	8.889 × 10^-6 ^(E_CD)

The hybrid model was also as accurate as the dynamic model in the case of a simple pathway with an underdetermined static module (Figure 2b). The maximum one-step errors at steady state were 5.049 × 10^-12 ^for metabolite concentrations and 2.837 × 10^-6 ^for reaction rates (Table 2). The time courses after a two-fold increase in the concentration of metabolite A were very similar between the dynamic and the hybrid model (Figure 3c and 3d). The maximum errors at the first integration step after the perturbation were 3.575 × 10^-7 ^for the metabolite concentrations and 0.00120 for the reaction rates.

**Table 2:** Errors between the dynamic model and the hybrid model of the pathway shown in Fig. 2b. The maximum errors were measured within one numerical integration step. "Perturbation" denotes whether the errors were measured under a steady-state condition (-) or after a two-fold increase of metabolite A (+)

	Perturbation	Maximum error (concentration)	Maximum error (reaction rate)
Boundary	-	5.049 × 10^-12 ^(F)	5.609 × 10^-12 ^(E_FG)
	+	3.575 × 10^-7 ^(C)	1.323 × 10^-7 ^(E_FG)
Static part	-	7.176 × 10^-15 ^(D)	2.837 × 10^-6 ^(E_CD, E_DF)
	+	1.192 × 10^-7 ^(D)	0.00120 (E_CD)

In contrast, the models did not agree as closely when (i) the static module involved enzymes of which the reactions were bottlenecks of dynamic behaviour, i.e. were not sufficiently susceptible to the boundary reaction rates, and (ii) a boundary reaction rate underwent a large change in response to changes in substrate concentrations. For example, the hybrid model of the hypothetical pathway with an over-determined static module exhibited approximately 10-fold higher one-step errors in the reaction rates of the static module when the rate constants of a boundary reaction E_BC were altered from k_f _= 0.01s^-1^, k_r _= 0.001s^-1 ^to k_f _= 0.1s^-1^, k_r _= 0.091s^-1^.

### Correlation between elasticity and errors

**Figure 4:**
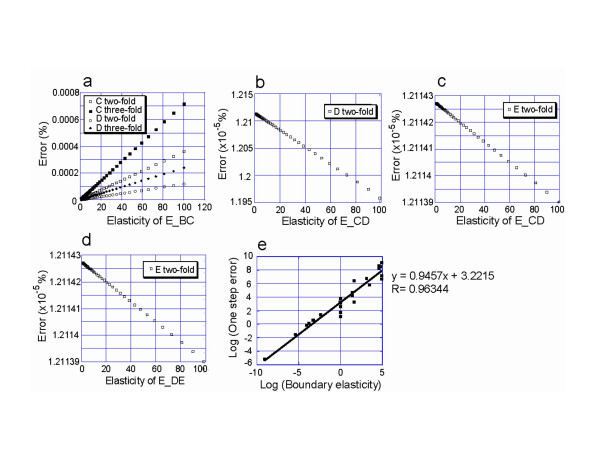
**Correlation between elasticity and error**. (a) The error between the hybrid model and the dynamic model was positively correlated with the elasticity of the boundary reaction. (b,c,d) The elasticity of the reactions replaced by a static module was negatively correlated with the error. (e) The correlation between error and elasticity was also observed in the glycolysis model.

Relationships between kinetic properties and one-step errors were examined in depth using a simple linear pathway at a steady state (Figure 2c) and 2a glycolysis model [13,20] (Figure 2d). Elasticity is a coefficient defined by metabolic control analysis. It represents the sensitivity of reaction rate to changes in substrate concentration (See Eq. (4) in Methods). The one-step errors of all the reactions in the static module (E_CD, E_DE, and E_EF) were proportional to the elasticity of the boundary reaction E_BC (Figure 4a). In addition, the errors of E_CD and E_DE were negatively correlated with their own elasticities (Figure 4b, c and 4d). It was also observed in the glycolysis model that the one-step errors of reaction rates in static modules are proportional to the elasticities of the boundary reactions (Figure 4e). These results were in good agreement with the implications derived from Eq. (2), that a static module should be composed of reactions with large elasticities and boundary reactions with small elasticities.

### Application to erythrocyte metabolism

**Figure 5:**
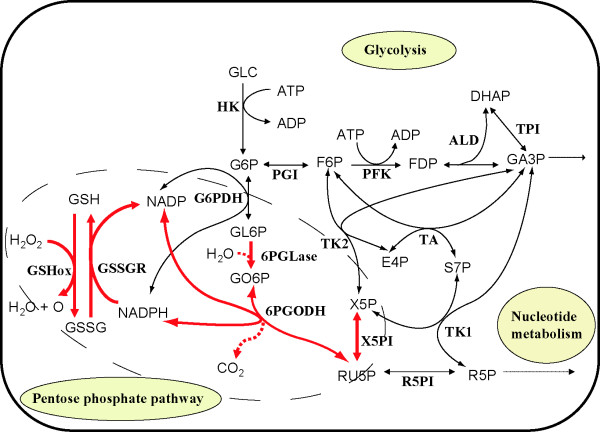
**A pathway map of the erythrocyte model**. The erythrocyte model contains 39 metabolites and 41 reactions (not all are shown here). The reactions represented by red arrows are placed in the static module of the hybrid model. The other reactions belong to the dynamic module. The abbreviations of metabolites and enzymes are described in Tables 4 and 5 in Additional file 1, respectively.

The same analysis was performed using an erythrocyte metabolism model [14] to evaluate the applicability of the hybrid method to more realistic and more complex pathways. A group of enzymes surrounded by glucose-6-phosphate dehydrogenase (G6PDH), transketolase I (TK1), transketolase II (TK2) and ribulose-5-phosphate isomerase (R5PI) was replaced with a static module (Figure 5) to verify the implications of Eq. (2), that a static module should be composed of reactions with large elasticities and boundary reactions with small elasticities. These enzymes were selected because they exhibit relatively small elasticity ratios (see Methods for definition) compared to others in this pathway. The static module is an over-determined system (eight metabolites and five reactions).

The hybrid and dynamic erythrocyte models yielded similar dynamics in response to a three-fold increase of FDP concentration (Figure 3e and 3f). The errors between the dynamic and hybrid models of the erythrocyte pathway were quantified by the procedure used for the hypothetical pathways. In a steady-state condition without an increase in FDP, the maximum error, 2.17 × 10^-4^, was observed in the reaction rate of 6-phosphogluconate dehydrogenase (6PGODH) (Table 3). (Note that this was true only when the gluconolactone-6-phosphate (GL6P) concentration was excluded. Owing to its small initial concentration (7.572 nM), the error in GL6P was sensitive to small changes and was associated with a large error of 0.00780.) The error in the 6PGODH rate remained the maximum error when the FDP concentration was perturbed.

**Table 3:** Comparisons of the dynamic model and the hybrid model of the erythrocyte pathway shown in Fig. 5. The maximum errors were measured within one numerical integration step. "Perturbation" denotes whether the errors were measured under a steady-state condition (-) or after a three-fold increase of FDP concentration (+).

	Perturbation	Maximum error (concentration)	Maximum error (reaction rate)
Boundary	-	7.796 × 10^-3 ^(GL6P)	1.555 × 10^-7 ^(R5PI)
	+	1.153 × 10^-7 ^(GL6P)	3.020 × 10^-5 ^(TK1)
Static part	-	1.111 × 10^-8 ^(GSSG)	2.170 × 10^-4 ^(6PGODH)
	+	4.282 × 10^-12 ^(GO6P)	2.170 × 10^-4 ^(6PGODH)

When the boundary reaction was relocated from G6PDH, which forms a bottleneck of dynamic response in a transient state and has low elasticity at steady state, to phosphoglucoisomerase (PGI), which has a larger elasticity, the time courses calculated by the hybrid model were different from those produced by the dynamic model.

## Discussion

In the simulation experiments using hypothetical pathways and an erythrocyte model, the discrepancies between the dynamic and the hybrid models were sufficiently small to prove that an MFA-based static module is capable of performing dynamic simulations as accurately as a kinetic model. The key idea behind our method is to distinguish between dependent and independent variables (reactions). Although independent reactions can be affected by other dependent/independent reactions through effectors such as ADP in the phosphofructokinase reaction, the time evolution of adjacent reaction rates are mainly determined by independent reactions which constitute bottlenecks of dynamic behaviour in the metabolic network. Therefore, static modules should consist of only such dependent reactions, whereas dynamic modules can include both independent and dependent reactions. Our hybrid method reduces the number of biochemical experiments required for dynamic models of large-scale metabolic pathways by replacing suitable enzyme reactions with a static module. The optimal conditions for this method are (a) a system with few bottleneck reactions in order to enlarge the static modules, (b) small fluctuations in the reaction rates in static modules, and (c) accurately identifiable bottleneck reactions. How can such enzymes be identified? One obvious criterion for the enzymes to be suitably modelled by a static module is not to incorporate a bottleneck reaction in a transient state. Thus, the enzymes should not reach the maximum velocity quickly or be restrained at lower activities by allosteric regulation. Although the model comprising dynamic and static modules as a whole can represent transient states, it is assumed that the reactions in the static modules achieve or nearly achieve steady states within one numerical integration step. The existence of one or more bottleneck reactions in the static module may cause inconsistencies, because the hybrid method solves algebraic equations for static modules under a steady state assumption, although metabolites will be accumulated or depleted in real cells. Therefore, bottleneck reactions must be excluded from static modules. Another situation that should be avoided involves reaction rates in static modules that are affected by changes in enzyme concentration, such as those caused by changing levels of transcriptional/ post-transcriptional control. Such reactions should be included in dynamic modules.

A similar cause of inconsistency is the reversibility of reactions. Since the hybrid method assumes that reactions in the static module are reversible, inclusion of an irreversible step may cause inconsistencies, particularly in the presence of a perturbation downstream of the irreversible step (data not shown).

The accuracy of the calculation can also be affected by a time lag. In the static module of the hybrid model, time lags between the upstream and downstream reactions are not represented because the boundary reactions affect all subsequent reactions in the static module within one integration step regardless of the number of enzyme reactions. Depending on the simulation time scale, the static module should be limited to minimize the influence of time lags. This influence can be estimated by the ratio of elasticities, which can be an important criterion for including a reaction in the static module.

The correlation between elasticity and one-step error (Figure 4) indicates that, to ensure the accuracy of the simulation, the static module of a pathway should include reactions with larger elasticities and should be surrounded by boundary reactions with small elasticities. A large elasticity indicates that the enzyme is capable of changing its reaction rate rapidly in response to changes in substrate concentrations [21]. The result shown in Figure 4 demonstrates that enzymes with large elasticity contribute to the accuracy of the static module. On the other hand, boundary reactions with small elasticities, large substrate concentrations and/or small reaction rates change their activities little in response to substrate concentrations over a short period of time; perturbations are thus dampened by boundary reactions before being transmitted to the static modules. As a result, the reaction rates in the static modules do not change much after perturbations. Such a moderate time evolution allows even reactions that are not very fast to realize a reaction-rate distribution, **v**, that can be calculated from **v **= **S**^#^**b **in as little as one numerical integration step. This allows the hybrid model to produce results that are in agreement with the dynamic model when the boundary reactions weaken perturbation.

The results we obtained when we relocated the boundary of the static module in the erythrocyte model support the importance of elasticity ratios. When G6PDH was included inside the static module, PGI became the new boundary reaction instead of G6PDH. The elasticity of PGI is large (elasticity = -452.496) compared to its neighbour G6PDH (elasticity = 0.0955). The relocated boundary is therefore composed of a pair of reactions that might produce unacceptable calculation errors, and in fact led to inconsistencies between the hybrid and dynamic models. Thus, the analytical conclusion presented in Eqs. (2) and (3) also holds for complex pathways, and elasticity provides a criterion for identifying groups of enzymes that can be approximated with sufficient accuracy by static modules. However, a large amount of experimental data is still required to determine the elasticities of all enzymatic reactions. In addition, the demarcation of the static module using elasticities determined by conventional biochemical experiments is unrealistic with respect to their throughput. Hence, the comprehensive determination of bottleneck reactions is the key task in the construction of large-scale metabolic pathway models using the hybrid method. Recent advances in flux measurement, quantitative metabolomics and proteomics allow large-scale measurement of flux distributions [22], intracellular metabolite concentrations and amounts of enzymes [23].

Recently, a method for high-throughput metabolomic analyses using capillary electrophoresis assisted by advanced mass spectrometry (CE-MS) and LC-MS/MS has been developed by the metabolomics group at our institute [24-27]. This technology allows us to determine the concentrations of more than 500 different metabolites quantitatively in a few hours. Furthermore, we are developing a method to calculate whole reaction rates of metabolic systems. This method has already achieved preliminary successes in determining the reaction rates of glycolysis in *E. coli *and human red blood cells. Pulse-chase analyses using ^13^C labeled molecules and the CE-MS/LC-MS high-throughput system have also been used successfully by the same metabolomics group to determine fluxes in the *E. coli *central carbon pathway.

Several approaches have been proposed to quantify elasticity and other coefficients of metabolic control analysis from experimental data such as flux rates, metabolite concentrations or enzyme concentrations [28-31]. Thus, the hybrid method, in combination with the 'omics' data of metabolism, enables a dynamic kinetic simulation of cell-wide metabolism.

## Conclusion

Using this hybrid method, the cost of developing large-scale computer models can be greatly reduced since precise modelling with dynamic rate equations and kinetic parameters is limited to bottleneck reactions. This drastically reduces the number of experiments needed to obtain the kinetic properties required for the dynamic simulation of metabolic pathways.

## Methods

### Calculation procedure

The hybrid method works within one numerical integration step as follows: (i) all the reaction rates in the dynamic module are calculated from dynamic rate equations (V_1_, V_2_, V_9_, and V_10 _in Figure 1); (ii) the reaction rate distribution in the static module (V_3_, V_4_, V_5_, V_6_, V_7_, and V_8_) is derived from the balance equation **Sv **= **b**, where **S **denotes the stoichiometric matrix, **v **the flux distribution, and **b **the rates of the dynamic exchange reactions at the system boundary (V_2_, V_9_, and V_10_) that are calculated in step (i); and (iii) the concentrations of the metabolites (X_1_-X_13_) are determined by numerical integration of the reaction rates calculated in steps (i) and (ii). All the reactions in the static module are assumed to be reversible.

The calculation of the reaction rate distribution in the static module is similar to that in the MFA method. The only difference is that the exchange reactions between the dynamic and static modules are represented by kinetic rate equations instead of constant fluxes. In this study, we term a dynamic exchange reaction of a static module a "boundary reaction". Dynamic boundary reactions provide quasi-dynamic changes in the reaction rate distribution in the static module. The reaction rate distribution in the static module is calculated at every integration step that refers to the boundary reaction rates, which are determined by concentrations of metabolites inside and outside the static module. The time evolution of the metabolite concentration in the static module is calculated at every integration step by numerical integration of the reaction rates as well as the metabolites in the dynamic module.

In step (ii), the Moore-Penrose pseudo-inverse is employed to calculate the reaction rate distribution of the static module at each numerical integration step. This should result in a smaller computational cost than linear programming, which is commonly used to determine the flux distribution of the underdetermined system. When the linear equation **Sv **= **b **is determined, **S**^#^, the Moore-Penrose pseudo-inverse of **S**, is identical to **S**^-1^, the inverse of **S**. Thus, the reaction rate distribution of the static module is solved uniquely as **v **= **S**^-1^**b**. If the equation **Sv **= **b **is over-determined, **v **= **S**^#^**b **provides the least squares estimate of the reaction rate distribution [32] which minimizes |**Sv-b**|^2^. Through this procedure, the error is distributed equally among the reaction rates of the static module.

In the case of an underdetermined static module, the solution was chosen from the solution space of the balance equation **Sv **= **b **to minimize the error of the ideal reaction rate distribution specified by the user. The optimal solution **v**_best _is represented in Eq. (1) below [see Supplementary Text 1 in Additional file 1 for the derivation]:-

**v**_best _= **i **+ **S**^# ^(**b **- **Si**)   (1)

where **v**_best _is the closest solution to the ideal reaction rate distribution i in the solution space [Figure 6 in Additional file 1].

### Evaluation of errors at steady state

To compare the accuracy of the hybrid method with the conventional dynamic kinetic method analytically, we first employed a pathway model comprising the three sequential reactions shown below. The whole pathway is assumed to be at a steady-state.



In the remainder of this report, a "dynamic model" refers to a metabolic pathway model that is represented by kinetic rate equations only. Let v_1_, v_2 _and v_3 _be the reaction rates of the three sequential reactions. In the hybrid model, the reaction rate v_2 _was represented as a static module of this pathway. When the concentration of metabolite A, the substrate of v1, is perturbed, the discrepancy between v2 in the hybrid model and v2 in the dynamic model is as described below [see Supplementary Text 2 in Additional file 1 for the derivation]:



where v_2d_, v_2k_, [A], [B], *ε*^v1^_A _and *ε*^v1^_B _denote the reaction rate v_2 _in the dynamic model, v_2 _in the hybrid model, concentration of metabolite A, concentration of metabolite B, elasticity of v_1 _with respect to metabolite A, and elasticity of v2, respectively. The variables with Δ are increments after a small time step Δt. The parameter p represents a ratio of the reaction rate in the static module to the influx, as in Δ*v*_2h _= *p*Δ*v*_1_. The ratio p is determined by the stoichiometric matrix of the pathway.

In Eq. (2), the left bracket term on the right-hand side indicates the magnitude of the perturbation transmitted to the static module. This term indicates that the error between the hybrid and dynamic models is proportional to the increment of metabolites and the elasticity of the boundary reactions. The right bracket describes the susceptibility of the reaction rate v_2 _to v_1_. When *ε*^v2^_B _satisfies the relationship below, v_2 _in the hybrid model exhibits identical time evolution to the dynamic model:



Since a small Δt (<<1.0s) is usually employed for accurate simulations of metabolic pathways, Eq. (3) implies that a reaction with large elasticity can be appropriately replaced by a static module.

For more complex pathways, such a theoretical analysis is not practical because large numbers of variables and parameters might impede clear discussions. Instead, simulation experiments were performed to compare the accuracy of hybrid models with dynamic models by numerical methods.

The accuracy of the hybrid model was evaluated numerically in comparison with a conventional kinetic model of the same metabolic pathway under two conditions: a steady-state condition and a time evolution after a two-fold increase of metabolites that are catalyzed by boundary reactions. The errors under steady-state conditions were employed as controls to evaluate discrepancies in dynamic behaviour. These computer simulations were performed using the E-Cell Simulation Environment version 1.1 or 3.1.102 for RedHat Linux 9.0/i386. The errors of reaction rates and metabolite concentrations were measured as below:-



where v_d _and v_h _denote either the reaction rates or the concentrations in the dynamic and hybrid models, respectively. The values of v_d _and v_h _were taken at the first numerical integration step, in which the concentration increase influences the initial steady-state values of the reaction rates and metabolite concentrations. In this article, this is termed "one-step error". We used one-step errors to represent the discrepancies between the two simulation methods in transient dynamics.

The one-step errors were evaluated using two simple pathways; the static module of one is determined, while the other is underdetermined (Figure 2a and 2b). All the reaction rates in these simple pathways were represented as v = k_f_[S]-k_r_[P] where v, k_f_, k_r_, [S], and [P] are a reaction rate, a forward rate constant, a reverse rate constant, a substrate concentration and a product concentration, respectively. In the pathway with the over-determined static module, the rate constants were k_f _= 0.05s^-1 ^and k_r _= 0.091s^-1 ^for E_CD and E_CE, k_f _= 0.1s^-1 ^and k_r _= 0.091s^-1 ^for E_DF and E_EG, and k_f _= 0.01s^-1 ^and k_r _= 0.001^-1 ^for the other reactions in the pathway of Figure 2a. The initial metabolite concentrations were 1.0 mM for A, B and C, and 0.5 mM for the other metabolites. Metabolite A was increased two-fold to evaluate the errors in transient dynamics. In the pathway with an underdetermined static module, the kinetic parameters were k_f _= 0.01s^-1 ^and k_r _= 0.001s^-1 ^for E_AB, E_BC, and E_FG; k_f _= 0.1s^-1 ^and k_r _= 0.098s^-1 ^for E_CD and E_DF; k_f _= 0.1s^-1 ^and k_r _= 0.097s^-1 ^for E_CE and E_EF; and k_f _= 0.1s^-1 ^and k_r _= 0.96s^-1 ^for E_CF. The steady-state flux distribution was employed for the ideal reaction rate distribution in the static module; the ideal reaction rates were 2 *μ*M/s for E_CD and E_DF, 3 *μ*M/s for E_CE and E_EF, and 4 *μ*M/s for E_CF. All the initial metabolite concentrations were 1.0 mM. The concentration of metabolite A was increased two-fold to evaluate the error.

### Correlation between elasticity and error

Elasticity is a coefficient used to quantify the sensitivity of the enzyme to its substrates and is defined as below in the context of metabolic control analysis [21]:



where [S] and v denote the substrate concentration and the reaction rate of the enzyme, respectively. Correlation between the one-step errors and elasticities of each enzyme at a steady state was examined using a linear pathway and a glycolysis model [13,20] (Figure 2c and 2d, respectively). In the linear pathway model, the reaction rate v is represented by the same equation as in the two hypothetical models above. The kinetic parameters were k_f _= 0.01s^-1 ^and k_r _= 0.009s^-1 ^for E_AB, E_BC, and E_FG and k_f _= 0.1s^-1 ^and k_r _= 0.099s^-1 ^for E_CD, E_DE, and E_EF. All the initial metabolite concentrations were 1.0 mM. The two rate constants of reactions E_BC, E_CD, E_DE, and E_DF were altered within the range 0.01<k_f_<1.0. The value of k_r _was determined to satisfy k_f_-k_r _= 0.01 to sustain the initial steady-state concentrations. The concentration of metabolite A was increased two-fold to evaluate the errors. For error measurements in the glycolysis model, each enzymatic reaction was replaced, one by one, with a static module. The substrate concentrations of the boundary reactions were increased three-fold.

### Application to erythrocyte metabolism

A cell-wide model of erythrocyte metabolism [14] was employed to evaluate the applicability of the hybrid method in a more realistic and complex pathway. This erythrocyte model reproduces steady-state metabolite concentrations similar to experimental data. The static region was determined using a ratio of elasticities as below:



where *ε*^b ^and *ε*^x ^denote the elasticities of a boundary reaction and of reaction X, respectively. All the elasticities of the model were calculated by numerical differentiation of each rate equation. A group of enzymes with small r values were regarded as appropriate candidates for inclusion in a static module. The concentration of fructose-1,6-diphosphate (FDP) was increased three-fold to measure the errors in dynamic behaviours.

## Competing interests

The author(s) declare that they have no competing interests.

## Authors' contributions

Yugi contributed to the development and implementation of the hybrid method into the E-Cell system, and developed methods for analyzing errors at a steady state. Nakayama provided the concept of hybrid method and directed the project. Kinoshita contributed to the development of simulation models and the analyses, and Tomita is a project leader.

## Supplementary Material

Additional File 1 Derivations of equations (Eqs. (1) and (2)), supplementary tables (Table 4 and Table 5) and figure (Figure 6). Click here for file
