# Supplementary material for: Hybrid dynamic/static method for large-scale simulation of metabolism
Source: Theor Biol Med Model. 2005 Oct 4;2:42. doi: 10.1186/1742-4682-2-42 (PMC1262783; doi:10.1186/1742-4682-2-42)
Supplement: Additional File 1 — Derivations of equations (Eqs. (1) and (2)), supplementary tables (Table 4 and Table 5) and figure (Figure 6). [file 1742-4682-2-42-S1.doc]

**Supplementary Information for “Hybrid dynamic/static method for large-scale simulation of metabolism”**

**(Yugi, K., Nakayama, Y., Kinoshita, A. and Tomita, M.)**

**Supplementary Text 1. Derivation of Eq. (1)**

Suppose that the reaction rate distribution **v** is represented as follows:

$$\mathbf{v}=\mathbf{i}+\mathbf{e}$$

where **i** denotes the ideal reaction rate distribution and **e** is the error between **v** and **i**. Substitution of this equation into the balance equation **Sv** = **b** yields

$\begin{matrix} \mathbf{S}(\mathbf{i}+\mathbf{e}) & = & \mathbf{b} \\ \mathbf{Se} & = & \mathbf{b}-\mathbf{Si} \\ \mathbf{e}_{\mathbf{min}} & = & \mathbf{S}^{\mathbf{\#}}(\mathbf{b}-\mathbf{Si}) \end{matrix}$

where **e**_min_ denotes the least error between **i** and **v** because the Moore-Penrose pseudo-inverse **S**^#^ provides the least-norm solution in the underdetermined system. Consequently, we obtain

$\begin{matrix} \mathbf{v}_{\mathbf{best}} & = & \mathbf{i}+\mathbf{e}_{\mathbf{min}} \\ & = & \mathbf{i}+\mathbf{S}^{\mathbf{\#}}(\mathbf{b}-\mathbf{Si}) \end{matrix}$
where **v**_best_ is the closest solution to the ideal reaction rate distribution in the solution space of **Sv** = **b** (Fig. 6).

**Supplementary Text 2. Derivation of Eq. (2)**

A theoretical analysis was performed employing a pathway model that includes three sequential reactions, as shown below:

$\to A\to^{v1} B\mathop{\Rightarrow}\limits^{v2} C\to^{v3} D\to$

The symbols v_1_, v_2_, and v_3_ denote the reaction rates of each step. The reaction rate v_2_ is represented by a static part in the hybrid model of this pathway. Suppose that the concentration of A is increased by $\Delta[A]$ at t = 0.

$$[A]\to[A]+\Delta[A]$$

Let $\Delta v_{1}$ be the increment of v_1_ in response to the increase of A. Then, $\Delta v_{1}$ is represented as the product of $\Delta[A]$ and the unscaled elasticity of v_1_ with respect to A ($E_{A}^{v1_{1}}$). The unscaled elasticity of a reaction rate v with respect to a metabolite S is defined as below:

$$\begin{matrix} E_{S}^{v} & = & \frac{\partial v}{\partial[S]} \\ & = & \frac{[S]}{v}\varepsilon_{S}^{v} \end{matrix}$$

where $\varepsilon_{S}^{v}$ denotes the corresponding scaled elasticity. For simplicity, we employed unscaled elasticity except in equation (S9).

$$v_{1}\to v_{1}+\Delta v_{1}$$

$\Delta v_{1}=\Delta[A]\cdot E_{A}^{v1_{1}}$ (S1)

In the next time step (t =$\Delta t$), the increase of v_1_ causes accumulation of the metabolite B.

$[B]\to[B]+\Delta[B]$

$\Delta[B]=\Delta v_{1}\cdot\Delta t$ (S2)

Substitution of equation (S2) with (S1) yields

$\Delta[B]=\Delta[A]\cdot E_{A}^{v1}\cdot\Delta t$ (S3)

Subsequently, v_2_ is activated by the accumulation of B.

$$v_{2d}\to v_{2d}+\Delta v_{2d}$$

$\Delta v_{2d}=\Delta[B]\cdot E_{B}^{v2}$ (S4)

Substituting equation (S4) with (S3), we obtain

$\Delta v_{2d}=\Delta[A]\cdot E_{A}^{v1}\cdot E_{B}^{v2}\cdot\Delta t$ (S5)

In the hybrid model, v_2_ is calculated as a product of v_1_ and $p$, which is a ratio of $\Delta v_{1}$ and $\Delta v_{2}$ determined by the stoichiometric matrix:

$\Delta v_{2h}=p\cdot\Delta v_{1}$ (S6)

Substitution of equation (S6) with (S1) yields

$\Delta v_{2h}=p\cdot\Delta[A]\cdot E_{A}^{v1}$ (S7)

From equation (S5) and (S7), the discrepancy of the dynamic and the hybrid model is

$\begin{matrix} \Delta v_{2d}-\Delta v_{2h} & = & \Delta[A]\cdot E_{A}^{v1}\cdot E_{B}^{v2_{1}}\cdot\Delta t-p\cdot\Delta[A]\cdot E_{A}^{v1} \\ & = & \Delta[A]\cdot E_{A}^{v1}(E_{B}^{v2_{1}}\cdot\Delta t-p) \end{matrix}$ (S8)

Replacing unscaled elasticities with scaled elasticities, we obtain

$\Delta v_{2d}-\Delta v_{2h}=\left(\Delta[A]\cdot\frac{v_{1}}{[A]}\cdot\varepsilon_{A}^{v1}\right)\cdot\left(\frac{v_{2}}{[B]}\cdot\varepsilon_{B}^{v2}\cdot\Delta t-p\right)$ (S9)

The discrepancy is equal to zero when the second bracket term of the right hand side is equal to zero.

$\frac{v_{2}}{[B]}\cdot\varepsilon_{B}^{v2}\cdot\Delta t-p=0$ (S10)

Transformation of (S10) yields the condition in which the elasticity of v_2_ produces zero error calculations by the hybrid method.

$\varepsilon_{B}^{v2_{1}}=\frac{[B]}{v_{2}}\cdot\frac{p}{\Delta t}$ (S11)

Thus, **v_best_** = **i** + **S^#^**(**b** - **Si**) represents the closest solution to the ideal reaction rate distribution **i**.

**Supplementary figures and tables**

**
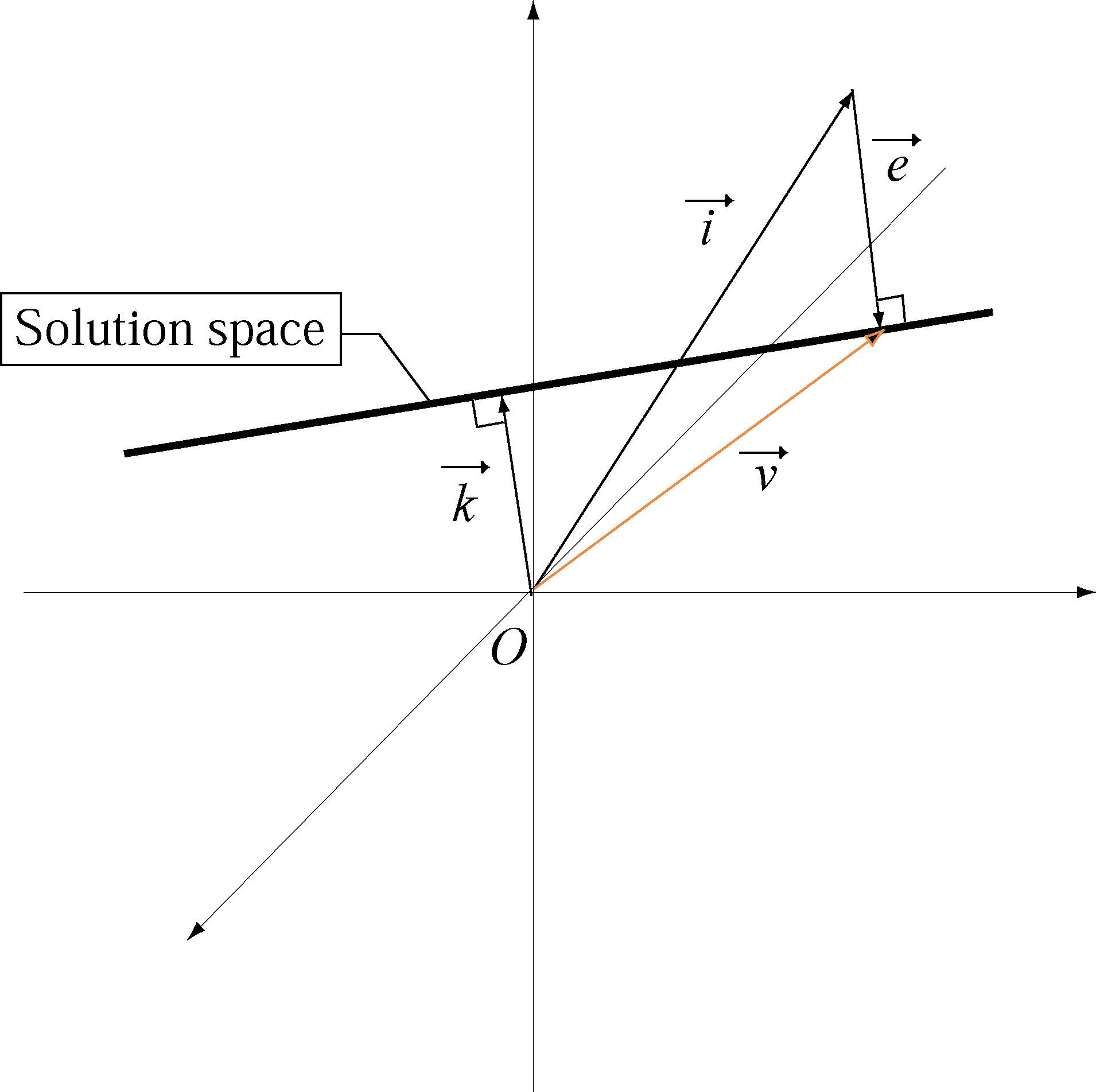
**

Figure. 6. An optimal solution of an underdetermined mass-balance equation **Sv** = **b** using the Moore-Penrose pseudo-inverse **S^#^**. Let **i** be the ideal reaction rate distribution vector, and **e** be the error vector representing the distance between **i** and the possible solution set. Substitution of **Sv** = **b** with **v** = **i** + **e** yields **Se** = **b** - **Si**. Since **S^#^** yields the least-norm solution (**k**)**,** the error vector **e** derived from **e_min_** = **S^#^**(**b** - **Si**) exhibits the least distance from **i** to the solution space. Thus, **v_best_** = **i** + **S^#^**(**b** - **Si**) represents the closest solution to the ideal reaction rate distribution **i**.

Table 4. Abbreviations of compound names.

| Abbreviation | Name of compound |
| --- | --- |
| 13DPG | 1,3-Diphosphoglycerate |
| 2PG | 2-Phosphoglycerate |
| 3PG | 3-Phosphoglycerate |
| DHAP | Dihydroxy acetone phosphate |
| f23DPG | 2,3-Diphosphoglycerate (free) |
| F6P | Fructose 6-phosphate |
| FDP | Fructose 1,6-diphosphate |
| G6P | Glucose 6-phosphate |
| GA3P | Glyceraldehyde 3-phosphate |
| LAC | Lactate |
| NAD | Nicotinamide adenine dinucleotide |
| NADH | Nicotinamide adenine dinucleotide |
| PEP | Phosphoenolpyruvate |
| Pi | Inorganic phosphate |
| PYR | Pyruvate |
| R5P | Ribose 5-phosphate |
| GL6P | Gluconolactone 6-phosphate |
| NADP | Nicotinamide adenine phosphate |
| NADPH | Nicotinamide adenine phosphate |
| RU5P | Ribulose 5-phosphate |
| X5P | Xylulose 5-phosphate |
| GO6P | Gluconate 6-phosphate |
| GSH | Glutathione (reduced) |
| GSSG | Glutathione (oxidized) |

Table 5. Abbreviations of the enzyme names.

| Abbreviation | Name of enzyme/reaction |
| --- | --- |
| 6PGLase | 6-phosphogluconolactonase |
| 6PGODH | 6-phospho-gluconate dehydrogenase |
| ALD | Aldolase |
| DPGase | Diphosphoglycerate phosphatase |
| DPGM | Diphosphoglycerate mutase |
| EN | Enolase |
| G6PDH | Glucose 6-phosphate dehydrogenase |
| GAPDH | Glyceraldehyde phosphate dehydrogenase |
| GSHox | Reduction processes consuming GSH |
| GSSGR | Glutathione reductase |
| HK | Hexokinase |
| LACtr | Lactate transport process |
| LDH | Lactate dehydrogenase |
| PFK | Phosphofructokinase |
| PGI | Phosphoglucoisomerase |
| PGK | Phosphoglycerate kinase |
| PGM | Phosphoglyceromutase |
| PK | Pyruvate kinase |
| R5PI | Ribulose 5-phoaphate isomerase |
| TA | Transaldolase |
| TK1 | Transketolase I |
| TK2 | Transketolase II |
| TPI | Triose phosphate isomerase |
| X5PI | Ribulose 5-phosphate epimerase |
